# Osteoblast behaviors on titania nanotube and mesopore layers

**DOI:** 10.1093/rb/rbw042

**Published:** 2016-12-24

**Authors:** Yan Zhang, Rong Luo, Jing Tan, Jianxin Wang, Xiong Lu, Shuxin Qu, Jie Weng, Bo Feng

**Affiliations:** Key Laboratory of Advanced Technologies of Materials, Ministry of Education, School of Materials Science and Engineering, Southwest Jiaotong University, Chengdu 610031, PR China

**Keywords:** nanotube, mesopore, osteoblast, adhesion, proliferation, mineralization

## Abstract

Titania nanotubes and mesopores with different diameter sizes were prepared by electrochemical oxidation of titanium. The responses of osteoblastic cells isolated from Sprague–Dawley rats to the nanotube and mesopore layers were investigated in sequential events of cell adhesion, morphology, actin cytoskeleton, proliferation, differentiation, and mineralization. Nano-structural features, especially diameters of the nanotubes and mesopores, obviously influenced on cell behaviors in the sequential events. The cells showed better proliferation and differentiation abilities on the specimens with the nanotubes and mesopores than on flat titanium disk. Higher levers of calcium mineralization were observed on the nanotube and mesopore layers. The cells adhered much faster onto the nanotubes with about 170 nm diameter and the mesopores with about 400 nm diameter than onto flat titanium disk and 50 nm nanotubes. There is an appropriate range of the tube/pore sizes, and in this present work, titania nantubes with 170 nm diameter is the best for enhancing functions of osteoblasts.

## Introduction

Recently, titania nanotubes on titanium surfaces have attracted much attention in biomaterial field. Anodized titania nanotubes with regular self-assembled array have shown good mechanical properties and excellent corrosion resistance, and they can firmly integrate to titanium substrate [1, 2]. After treatment by NaOH solution, the nanotubes were bioactivated and could induce growth of nano-hydroxyapatite (HA) in a simulated body fluid [3]. HA coatings also spontaneously formed on the nanotubes without any surface treatment in a biomimetic solution and had higher bond strength of the coatings to substrate [4]. Nanotubes on titanium surface would be favorable to adhesion and proliferation of osteoblasts, and heat-treated nanotubes were more effective than that without heat treatment. Moreover, the filopodia of propagating osteoblasts could go inside the vertical nanopores of titania nanotubes, which would promote bone growth and enhance integration to materials [5, 6]. In our previous work, we showed that nanostructured topologies can improve the proliferation, differentiation, and development of the osteoblastic phenotype [7]. In addition, mesoporous oxide layer can be obtained by microarc oxidation of titanium, and led to better cell biocompatibility compared to flat titanium [8–10].

Implant surface-specific cellular responses involve behaviors of a complex biological system, which include protein adsorption, receptor-ligand binding, and signal transduction [11]. So, the relationships between surface nano-features and cellular responses are quite complicated and far from being well understood. A precise understanding of cell–implant interactions in the peri-implant region is essential for designing surface-nano-structural biomaterials.

In the present study, we investigated behaviors of osteoblastic cells isolated from Sprague–Dawley rat on nanotube and mesopore layers with different tube/pore diameter sizes. We focused on the early responses such as cell adhesion, morphology, actin cytoskeleton, and proliferation to understand subsequent cellular development on nano/meso-structural titanium surfaces.

## Materials and methods

### Specimen preparation

Commercially pure titanium disks with 10 mm diameter and 1.5 mm thickness were ground with 600, 1000, and 1500-grit SiC papers, then were washed ultrasonically in turn with acetone, ethanol, and deionized H_2_O. After that, they were chemically cleaned in 6.0 M HNO_3_ and 1.0 M HF, washed ultrasonically in deionized H_2_O again, and dried. Pretreated titanium disks were used for electrochemical oxidation and as control (0 nm).

The preparation of titania nanotubes has been described in detail elsewhere [12]. In chief, anodic oxidization was carried out using a direct current voltage power (DC, DHEP 600V/6A). Electrolyte was a mixture of 0.15 mol/l HF and 2 mol/l H_3_PO_4_ solutions. Specimens with the smallest nanatube diameter were prepared by anodic oxidation at 10 V for 1.5 h. Large nanatubes were obtained by two-step anodic oxidation at 20 V and then 25 V for 1.5 h each step. Anodized specimens were heat-treated at 500°C for 3 h to crystallize the as-deposited amorphous-structural titania nanotubes. Titania mesopore specimens were fabricated by microarc oxidation at 150 V for 5 min in 1.0 M H_2_SO_4_ electrolyte [9].

### Characterization of oxide layers

Morphologies of the nanotubes and mesopores were observed using a scanning electron microscope (SEM, FEI Quanta 200). Crystal structures of oxide layers were analyzed with an X-ray diffractometer (XRD, X’Pert Pro MPD). Surface roughness of specimens was detected with a surface roughometer (JB-3C, Shang Hai), and at least four different positions on each specimen were tested to obtain the profile arithmetic average error (Ra). The water contact angles on specimen surfaces were measured using a goniometer (KrussGmbH DSA 100, Germany) followed by image processing of sessile drop with DSA 1.8 software. At least, five droplets were tested on different positions of each specimen surface.

### Cell culture

Primary osteoblasts isolated from Sprague–Dawley rats were provided by West China College of Stomatology (Chengdu, China). The cells were cultured with α-Minimum Essential Medium (α-MEM, Gibco, USA) supplemented with 15% (vol.%) fetal bovine serum (Yuan Heng Sheng Ma, China), 200 units/ml penicillin and 200 units/ml streptomycin. The cells were incubated under 37°C and 5% CO_2_ environment. When passaged to the third generation, the cells were seeded at about 3.8 × 10^4^ cells/cm^2^ onto specimens in 24-well polystyrene plates. To induce spontaneous differentiation into mature osteoblasts, a differentiation medium was prepared by adding 50 μg/ml ascorbic acid and 10 mM β-glycerophosphate to the growth medium.

### Cell morphology

After culture for 2, 6, and 12 h, the specimens were rinsed with phosphate buffer solution (PBS) three times and fixed with 2.5% glutaraldehyde. After that, they were dehydrated with a graded series of ethanol (30, 50, 70, 90, 95, and 100 vol%) and dealcoholizated with a graded series of ethylacetate (50, 70, and 100 vol%). These specimens were then dried in vacuum drying oven at 25°C and 1.325 × 10^−^^3^ MPa. Finally, they were sputter-coated with gold for SEM examination.

### Actine staining

Actine staining was performed using phalloidin coupled to fluorescein-isothyo-cyanate (Phalloidin-FITC, Sigma Aldrich). After culture for 6 h, 12 h, 1 day, and 2 days, the cells on specimens were washed with PBS and then fixed with 3.7% formaldehyde in PBS for 5 min. Subsequently, the cells were washed thoroughly in PBS, dehydrated with acetone, permeated with 0.1% Triton X-100 in PBS, and rinsed again in PBS. About 50 μg/ml Phalloidin-FITC in PBS was used for cell stain at room temperature. After 40 min, the cells were washed several times with PBS to remove unbound phalloidin conjugate. Finally, the cells were viewed with a fluorescent microscope (Leica Microsystems Wetzlar GmbH, Germany) at 495 nm wavelength.

### Alamar blue assay

Cellular proliferation was determined by an alamar blue assay. After culture for 12 h, 24 h, 2 days, and 7 days, the specimens with cells removed from the old culture were washed with PBS, and incubated again in fresh culture medium containing 10% alamar blue (Biosource, Camarilo, CA) under 37°C and 5% CO_2_ environment for 3 h. The blank reference was taken from wells without cells, and also cultured with the fresh culture medium. Optical absorbance of the culture medium from each well was measured by a microplate reader (μ-Quant, Bio-Tek Instruments, USA) at 570 nm wavelength. The absorbance was considered to have a linear relationship with the number and activity of cells. The test was repeated three times.

### Alkaline phosphatase activity

Alkaline phosphatase (ALP) is an early marker of cell differentiation and relates to the production of a mineral matrix. 4-Nitrophenyl phosphate was used as a substrate for determining the ALP activity. After culture for 2, 7, and 13 days, the culture medium was discarded and the specimens were washed with PBS for three times. The cells on the specimens were lysed with 1% TritonX-100 (Sigma-Aldrich Inc., St. Louis, MO) in culture plates, and then they were put into a refrigerator at −20°C. After three times freezing–thawing cycles, 50 μL lysis solution was mixed with 200 μL of 10 mM 4-nitrophenyl phosphate (4-NPP, Sigma-Aldrich Inc., St. Louis, MO) solution in each well of 96-well plate. The enzymatic reaction was stopped by adding 100 μl of 0.5M NaOH. ALP activity was measured by a microplate reader at 405 nm wavelength. The measurement was repeated three times.

### Mineralized nodule formation

Mineral matrix was stained for calcium by the Alizarin Red-S (AR-S, Alizarin Red-S Kit, Genmed) staining method operating as the kit specification. The blank reference was taken from the cells seeded on plate wells without specimen. Morphologies of the mineral matrix and calcium nodules were observed under an optical microscope. After culture for 12, 15, and 19 days, the cells were washed with PBS, and then PBS was sipped up. The specimen surfaces were washed three times with 1 mol/l NaOH for lysing the cells. In the same way, 1 mol/l HCl was added to dissolve the mineral matrix and calcium nodules for three times. All lysates from the same specimen were collected and homogenized. Calcium concentrations of the lysates were detected with an atomic absorption spectrophotometer (F-AAS, Z-5000) to calculate calcium contents mineralized on the specimens. The test was performed in triplicate. Analysis of calcium nodules was carried out using a SEM equipped with an energy dispersive X-ray spectrometer (EDS).

## Results

### Titania nanotubes and mesopores on titanium surfaces

**Figure 1:**
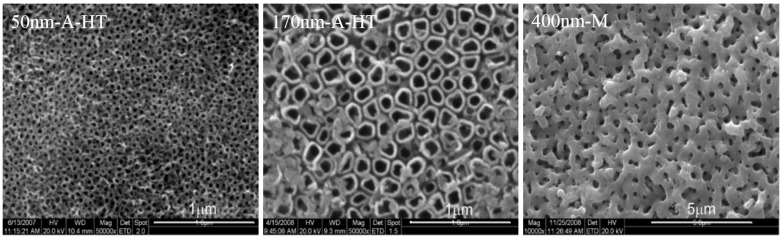
SEM Micrographs of titania nanotubes and mesopores on titanium surfaces (a) At 10 V for 1.5 h and then heat-treatment; (b) at 20–25V for 1.5 h and then heat-treatment; (c) at 150 V for 5 min

Nanotubes on titanium surfaces with average inner diameters of about 50 nm (50 nm-A) and about 170 nm (170 nm-A) were obtained by anodic oxidation at 10 V and 20—25 V, respectively, as shown in Fig. 1a and b. Mesopores on the surfaces with about 400 nm inner diameter (400 nm-M) formed after microarc oxidation at 150 V (Fig. 1c).

### Surface crystal structure, roughness, and hydrophilicity of specimens

**Figure 2:**
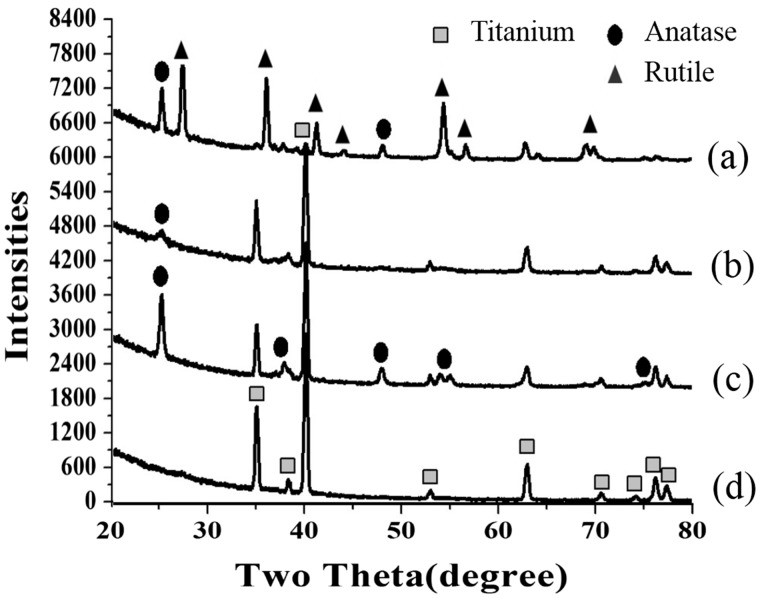
XRD patterns of nanotube arrays (a) 400 nm-M, (b) 170 nm-a-HT, (c) 50 nm-a-HT, and (d) 0 nm

The titanium oxide film formed naturally in air is dense and stable anatase titania with a thickness of about a few nanometers [13, 14]. As shown in Fig. 2, amorphous titania of 50 nm-A and 170 nm-A surfaces after heat-treatment transformed into anatase phase (50 nm-A-HT and 170 nm-A-HT). The surface of 400 nm-M consisted mainly of rutile phase in addition to anatase phase.

Roughness (Ra) of 0 nm, 50 nm-A-HT, 170 nm-A-HT, and 400 nm-M were 137 ± 6.27 nm, 133 ± 2.33 nm, 211 ± 5.66 nm, and 184 ± 4.62 nm, respectively. The surface of 170 nm-A-HT showed the largest roughness.

Water spread and formed a contact angle of 0º when was dropped onto the surfaces of 170 nm-A-HT and 400 nm-M, which exhibited their super-hydrophilicity. The contact angles on 0 nm and 50 nm-A-HT were 42 ± 2.7º and 6.2 ± 0.3º, respectively. Electrochemical oxidation and heat treatment increased hydrophilicity of titanium surfaces.

### Cell morphology on specimen surfaces

**Figure 3:**
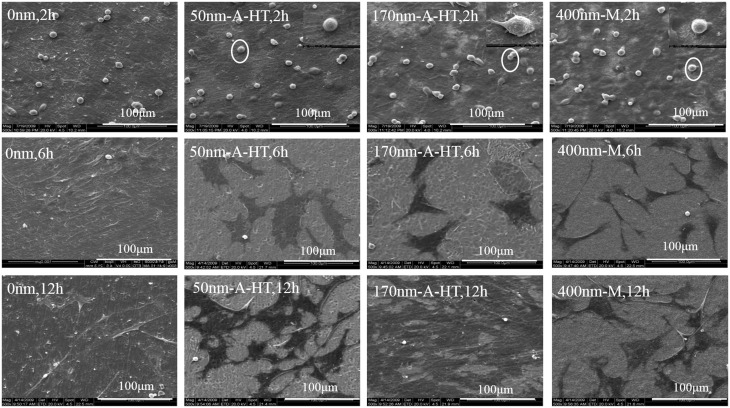
SEM micrographs of osteoblastic cells on the specimens after culture for 2, 6, and 12 hMagnified pictures were the place marked out by small circles

As shown in Fig. 3, after 2 h, the cells on 0 nm and 50 nm-A-HT were stereoscopic and showed round configurations with less pseudopodia compared with that on 170 nm-A-HT and 400 nm-M. Half of the cells on 170 nm-A-HT and 400 nm-M were oval configurations with pseudopodia growing laterally.

After 6 h, the cells on the specimens with the nanotubes and mesopores were flat and began to form an irregular border with pseudopodia spreading sufficiently. The cells on 0 nm were spindle-shaped configuration and stereo without pseudopodia growing laterally. The diameter of the nanotubes and mesopores significantly influenced cell morphologies.

After 12 h, the cells on 50 nm-A-HT, 170 nm-A-HT and 400 nm-M sufficiently spread and adhered to substrates with filopodia mutual connection. On 170 nm-A-HT surfaces, cells significantly connected and overlapped and the surfaces were fully covered by a continuous sheet of cells. In addition, the cells on 0 nm still kept spindle-shaped configuration only with pseudopodia growing laterally.

The specimens with the nanotubes and mesopores were more favorable to cell adhesion and growth than flat titanium disk. The diameter of the nanotubes and mesopores could influence cell adhesion and growth abilities. The cells adhered to 170 nm-A-HT and 400 nm-M at much faster speeds than to others. It took about 12 h for a noticeable adhesion and propagation of cells on 0 nm.

### Cytoskeleton by fluorescent staining

**Figure 4:**
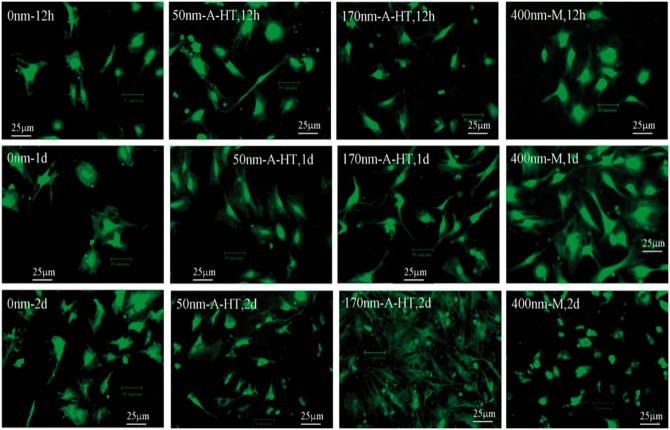
Fluorescence micrographs of osteoblastic actin cytoskeleton on the specimens after culture for 12 h, 1 day, and 2 days

Figure 4 shows the fluorescence images of osteoblastic actin cytoskeletons on the specimens. No significant difference in the actin cytoskeletons was found among the specimens with the nanotubes and mesopores after 12 h culture. Osteoblastic actin fibers on the specimens were elongated and stretched across the whole cells. The fibers around the cells were arranged radially. After 1 day, the cytoskeletons on the nanotubes and mesopores showed larger difference. The fibers on 50 nm-A-HT surface showed inhomogeneous distribution compared with those on 170 nm-A-HT and 400 nm-A-HT surfaces. The fibers on 170 nm-A-HT surface were elongated and showed radial shape. On 400 nm-M surface, the fibers also elongated and extended sufficiently. After 2 days culture, the surface of 170 nm-A-HT was almost fully covered by cells and fibers were more stretched than others. However, on 400 nm-M surfaces, the fibers showed neither elongation nor extensions on the surface (Fig. 4) .

### Activity, proliferation, and differentiation of osteoblasts

**Figure 5:**
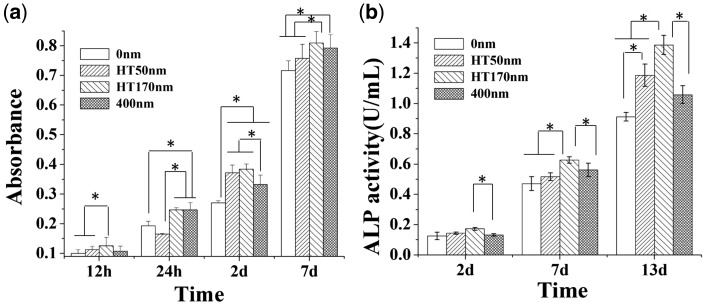
Alamar blue assay (a) and ALP activity (b) of osteoblasts on the specimens after culture (*n* = 3, *P < 0.05)

The diameters of the nanotubes and mesopores influenced the number (Fig. 5a) and ALP activities (Fig. 5b) of the cells. In culture period of 2 days, the cell number on 400 nm-A-HT was lower than those on 50 nm-A-HT and 170 nm-A-HT. And ALP activities on the nanotubes and mesopores were slightly higher than that on 0 nm. After 7 days, 170 nm-A-HT had the maximum number and ALP activity of the cells. When culture period extended to 13 days, cells on the nanotublar and mesoporous specimens all exhibited higher ALP activity. That is, the specimens with nano/meso-structure, especially 170 nm-A-HT, had better ability of proliferation and differentiation than flat titanium disk.

### Mineralization and calcium concentrations

**Figure 6:**
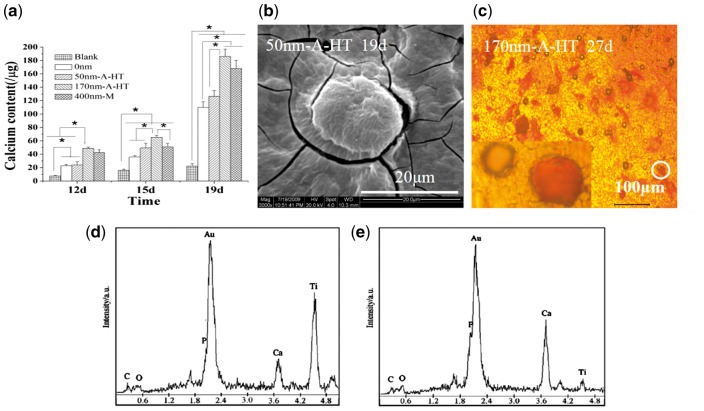
Contents of calcium mineralized on specimens after 12, 15, and 19 days culture (a). SEM micrographs of a calcium nodule (b) and AR-S staining of calcium deposited on 170 nm-a-HT (c). EDX spectra of calcium nodules at the edge of nodules (d) and on the top of nodules after 19 days culture (e), (*n* = 3, *P < 0.05)

Figure 6a indicates that calcium contents on the specimens increased quickly as prolong of culture time. The blank had the lowest calcium content, next the 0 nm. The highest content existed on 170 nm-A-HT. Stereoscopic and round-shaped calcium nodules were observed after about 19 days culture, as shown in Fig. 6b. The ratio of calcium to phosphorus at the edge of the nodules was less than one (Fig. 6d), which was caused by the generic calcium deposition in cytoplasm. The ratio at the top of the nodules was more than one (Fig. 6e), which was attributed to congregation of calcium salt on the extracellular. After 27 days culture, calcium nodules could be observed by AR-S stain and the most calcium nodules appeared on 170 nm-A-HT as shown in Figure 6c.

## Discussion

The cells preferred to adhere and propagate on the specimens with the nanotubes and mesopores. A similar result was reported about the adhesion of MC3T3-E1 cells on pretreated titania nanotubes [6]. The nanotubes and mesopores significantly increased surface roughness, which provided more surface area for osteoblastic early adhesion and propagation [15, 16]. The roughness promised the deposition of extracellular matrix (ECM) in an appropriate geometrical orientation which gives cell adhesion receptors access to specific sites in ECM molecules, thereby including cell spreading [17]. Surface nano-structure resulted also in high hydrophilicity. Cells adhered, spread, and grew more easily on hydrophilic substrates than on hydrophobic ones [16, 18]. Moreover, the cell adhesion and propagation related to the diameters of the nanotubes and mesopores. The cells adhered at much faster speeds to 170 nm-A-HT and 400 nm-M with super-hydrophilicity than to 50 nm-A-HT. In addition, the anatase-structured titania in the nanotube layers was beneficial to initial adhesion and spreading of osteoblasts-like cells [19]. The surface of flat titanium disk (0 nm) was only a thin dense and passivated titania layer.

The diameters of the nanotubes and mesopores influenced on the cellular morphologies and cellular actin cytoskeletons. It has been reported that bone cells took the topography of substrates for orientation and migration, a process known as contact guidance [20]. Up to now, the mechanism of how cells detect and respond to nano-topography is not quite clear. One possible mechanism is that nanometer-scale topology could influence the cytoskeleton forming and the interfacial tension of membrane receptor, and subsequently change cellular signaling pathways [21, 22]. It was found that arrangement of cytoskeleton changed when fibroblasts responded to nano-columns and the control [23]. In this work, 1 day culture later, the cellular actin cytoskeletons on the specimens appeared obvious difference. The nanotubes and mesopores with different diameters should result in different surface energy of the specimens. The specimens with high surface energy could strong interact with membrane receptor of cells, which would modulate cytoskeleton formation and change cellular morphologies. The 170 nm-A-HT had the highest surface energy above 72.80 mJ/m^2^. Although 400 nm-M had an approximately equal surface energy of about 72.73 mJ/m^2^, rutile phase in its oxide layer probably resulted in a decrease of its cytocompatibility compared with anatase.

It is accepted that nano-topology had great influence on osteoblastic proliferation and differentiation [24, 25]. This work indicated that the cells appeared better proliferation and differentiation on the nanotubes and mesopores compared to on flat titanium disk. And there was a size regime for regulating the cell behaviors of initial adhesion, growth, and ALP activity of osteoblasts. Oh *et al.* [26] suggested that cells adhered more easily onto the smaller diameter nanotubes instead of the larger nanotubes since more proteins aggregated on the smaller diameter nanotubes. However, our results showed that large diameter, 170 nm nanotubes could provide better microenvironment for cell growth. The nano-topography of the specimens significantly improved proliferation and differentiation with the filopodia of the growing cells going into the nanotubes and mesopores, producing a network structure. These abilities increased with an increase of the nanotube diameters in the sequence of 0 nm, 50 nm, and 170 nm. The nanotubes with larger diameters have larger space, which can lead filopodia to much easier ingrowth. In addition, our previous work has indicated that the specific surface area of the nanotube layers increased with an increase of the diameters in the present size range [27]. Generally, greater specific surface area leads to higher surface energy and corresponds to higher surface activity, which should be beneficial to cell adhesion and proliferation [16]. The trend of ALP activity seemed to correspond with the elongation trend, which may result in cytoskeletal stress and selective differentiation into osteoblast-like cells. The cells seeded on 170 nm-A-HT had a greatest elongation trend and ALP activity. However, 400 nm-M showed lower proliferation and differentiation than 170 nm-A-HT. As mentioned above, the former was mainly rutile titania, whereas the latter was all anatase. Anatase could enhance cell differentiation by enhancing osteogenic gene expression [28, 29]. Besides, 170 nm-A-HT showed the largest surface roughness. In fact, the roughness of nanotube layers mainly depends on the diameter of nanotubes. Consequentially, the influence of the surface roughness on cell behaviors corresponds to that of the nanotube diameter.

Calcium deposited in cell matrix reflects ossification level of osteoblasts. Calcium contents deposited on the nanotubes and mesopores were higher than that on flan titanium disk and the blank. Many calcium nodules formed on 170 nm-A-HT but few calcium nodules on the blank after 27 days culture. Existence of the nanotubes and mesopores shortened the time of osteoblastic ossification and 170 nm-A-HT had the highest ability of bone-forming.

The mechanism of calcium deposition is far from clear, which should relate to the increase of local ALP activity. The enzyme can hydrolyze organic phosphate and increase local phosphoric acid, which is prone to form deposition of calcium phosphate [30–32]. Variation of calcium deposition had the same trend as alteration of the ALP activity. That is, the diameters of the nanotubes and mesopores regulated on the calcium deposition by adjusting ALP activity.

## Conclusions

The diameter of the titania nanotubes and mesopores on titanium surfaces influenced on sequential events of osteoblastic cells, including adhesion, morphology, actin cytoskeleton, proliferation, differentiation, and mineralization of cellular calcium. Cells adhered much faster on 170 nm nanotube layer and 400 nm mesopore layers than on flat titanium disk and 50 nm nanotube layer. The 170 nm nanotubes showed the best abilities in proliferation, differentiation and mineralization. The nano/meso-structures enhanced functions of osteoblasts. In this present work, an appropriate nano-structure feature on titanium surfaces was titania nanotubes with 170 nm diameter and anatase crystal in terms of bone-forming.
